# Investigating the foreign body response and regenerative mechanisms in medical-grade polycaprolactone scaffold guided breast reconstruction in a porcine model

**DOI:** 10.3389/fbioe.2026.1788533

**Published:** 2026-06-17

**Authors:** Leopold Klein, Ronja Finze, Jan Janzekovic, Matthew Cheng, Siamak Saifzadeh, Owen Ung, Michael Wagels, Dietmar W. Hutmacher, Flavia Medeiros Savi

**Affiliations:** 1 Department for Medical Technologies and Regenerative Medicine, Institute of Biomedical Engineering, Eberhard Karls University Tübingen, Tübingen, Germany; 2 Centre for Biomedical Technologies, School of Mechanical, Medical and Process Engineering, Queensland University of Technology, Brisbane, QLD, Australia; 3 Department of Hand, Plastic and Reconstructive Surgery, BG Trauma Center Ludwigshafen, University of Heidelberg, Heidelberg, Germany; 4 Department of Plastic and Reconstructive Surgery, Princess Alexandra Hospital, Woollongabba, QLD, Australia; 5 Medical Engineering Research Facility, Queensland University of Technology, Chermside, QLD, Australia; 6 MNHHS Comprehensive Breast Cancer Institute, Royal Brisbane and Women’s Hospital, Herston, QLD, Australia; 7 Herston Biofabrication Institute, Metro North Hospital and Health Service, Herston, QLD, Australia; 8 Medical School, The University of Queensland, Herston, QLD, Australia

**Keywords:** 3D printing, biocompatibility, breast reconstruction, IHC, polycaprolactone, porcine model, scaffold guided tissue regeneration

## Abstract

To enhance the clinical use of scaffold-guided breast reconstruction (SGBR), the porcine model provides a reliable means of assessing scaffold biocompatibility and tissue regeneration. Understanding the immunological processes triggered by the implantation of medical-grade polycaprolactone (mPCL) scaffolds is essential for elucidating the underlying biology and optimizing the regenerative potential of tissue engineering and regenerative medicine (TE&RM) technologies. This observational work aims to characterize these *in vivo* processes through systematic, comprehensive immunohistochemical (IHC) analysis based on two preclinical large-animal studies. Key emphasis is placed on evaluating the interplay between immune cell-mediated foreign body response (FBR) and the cellular mechanisms required for successful soft-tissue regeneration, remodelling, and scaffold degradation. Findings indicate that the porcine model provides valuable insights into extracellular matrix (ECM) formation, the interactions between immune cells and the implant surface, and their spatial distribution within the scaffold’s porous architecture. This study further reflects the complexity and spectrum of macrophage phenotypes present 12 months after implantation, exhibiting distinct spatial distributions relative to the scaffold surface, consistent with their established roles in inflammation, tissue regeneration, and remodelling. It also emphasizes the importance of both cellular components and ECM deposition that coexists and may interact to create a distinct microenvironment that varies with local scaffold topography, resulting in location-dependent differences in cellular composition and ECM characteristics relevant to tissue reorganization and homeostasis. Furthermore, this research suggests that extensive neoangiogenesis and vascular remodelling throughout the scaffold architecture are essential for maintaining tissue viability and promoting autologous fat tissue regeneration within the scaffold’s large, fully interconnected pores. These factors are critical for understanding *in vivo* tissue regeneration following mPCL scaffold implantation and for advancing the translation of SGBR into clinical practice.

## Introduction

In recent years, the fields of tissue engineering and regenerative medicine (TE&RM) have aimed to regenerate soft tissue suitable for the reconstruction of clinically relevant defects (Donnely et al., 2020). Large-animal models, such as the porcine model, are increasingly used to address relevant tissue-regeneration volumes in preclinical settings, given their suitability for soft-tissue regeneration (Lunney et al., 2021; Patrick et al., 2008).

A recent review highlighted various TE&RM approaches for regenerating soft tissue in breast reconstruction, underscoring the diverse nature of these strategies and their potential in improving breast reconstruction outcomes (Berkane et al., 2024). One effective method is Scaffold-Guided Breast Reconstruction (SGBR), which aims to regenerate host tissue using additively manufactured, highly porous, biodegradable scaffolds, for instance, composed of medical-grade polycaprolactone (mPCL). When coupled with autologous fat grafting (AFG) or platelet-rich plasma (PRP), this method aims to stimulate a regenerative response by using the host as a bioreactor, thereby promoting tissue formation and maturation. At the same time, the scaffold gradually degrades over several cycles of tissue remodelling, leaving no foreign body residue and attempting to avoid the side effects and limitations associated with the current breast reconstruction paradigm (Chhaya et al., 2016; Cheng et al., 2021; Janzekovic et al., 2022; Cheng et al., 2024). Despite its name, SGBR does not seek to replicate the anatomical structure of the breast, which comprises glandular tissue and its associated functions. Instead, it focuses on regenerating tissue composed of an adipose and connective component that closely resembles native breast tissue, minus the glandular component, in both form and tissue smoothness.

Understanding the precise spatial cellular processes involved in SGBR is *a conditio sine qua non*, as it allows for the guidance of the complex biological process towards the desired outcome - soft tissue regeneration for breast reconstruction. A key advantage of investigating tissue regeneration and remodelling within SGBR using a porcine model, as opposed to clinical studies involving human patients, is the ability to explant the regenerated tissue at defined endpoints, enabling the visualization of *in vivo* tissue regeneration processes through immunohistochemical (IHC) and histological analyses (De Matos et al., 2010). The implantation of a medical device, such as an mPCL scaffold in the context of scaffold-guided regeneration triggers a cascade of complex cellular and extracellular matrix (ECM) processes that ultimately lead to tissue formation (Finze et al., 2023).

This work aims to demonstrate the efficacy of IHC for evaluating tissue regeneration in a porcine model using mPCL scaffolds. It characterizes immune cells, vascular components, ECM, and adipose tissue, thereby validating specific IHC markers as a foundation for future preclinical research on soft-tissue regeneration in pigs.

## Results

The research findings are derived from a comprehensive IHC analysis of regenerated tissue within highly porous mPCL scaffolds (Figures 1, 2) over 12 months, encompassing two scaffold volumes (100 mL in study 1 and 400 mL in study 2). The following examination delineates commonalities in tissue composition and the spatial distribution of cells and ECM across both studies and all experimental groups, thereby highlighting the characteristic tissue response to implantation of mPCL scaffolds in the context of SGBR.

**FIGURE 1:**
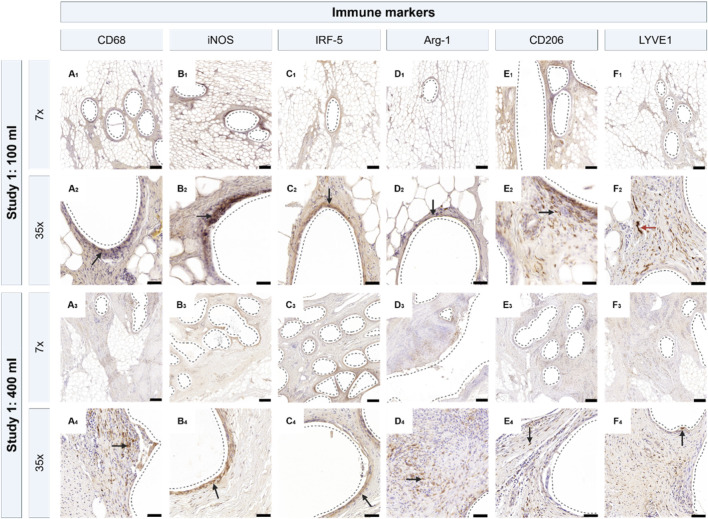
Immunohistochemical visualisation of the immune response to 100 ml (study 1) and 400 ml (study 2) volume porous mPCL breast scaffolds (mPCL scaffold only group) 12 months after implantation in a porcine model. The accumulation of immune cells, particularly macrophages and FBGCs, on and around the mPCL scaffold is notably marked by CD68 (**A_1–4_**, black arrows). Immunohistochemical examination reveals the spatial distribution of iNOS and IRF-5 labelled M1 macrophages, and M2 macrophages, which are identified by the expression of Arg-1 and CD206, in distinct locations. iNOS and IRF-5 expressing macrophages and FBGCs form the first cell layer around the scaffold struts and come into direct contact with its surface (**B_1–4_, C_1–4_** black arrows). Arg-1 and CD206 positive cells are situated adjacent to the M1 macrophages and FBGCs and can be found in the second layer, as well as disseminated within the connective tissue in between mPCL struts (**B_1–4_, C_1–4_** black arrows). LYVE1 highlights both macrophages and FBGCs adjacent to the scaffold surface (F_4_, arrow), as well as macrophages within the surrounding connective tissue at a greater distance from the scaffold (F_2_, black arrow). Newly formed lymphatic vessels within the regenerated tissue are also depicted by LYVE-1 positive lymphatic endothelial cells (F_2_, red arrow). mPCL struts are delineated with a grey dashed line. Scale bars: 200 µm **(A–F**_**1,3**_ **)** and 50 µm **(A–F**_**2,4**_ **)**.

**FIGURE 2:**
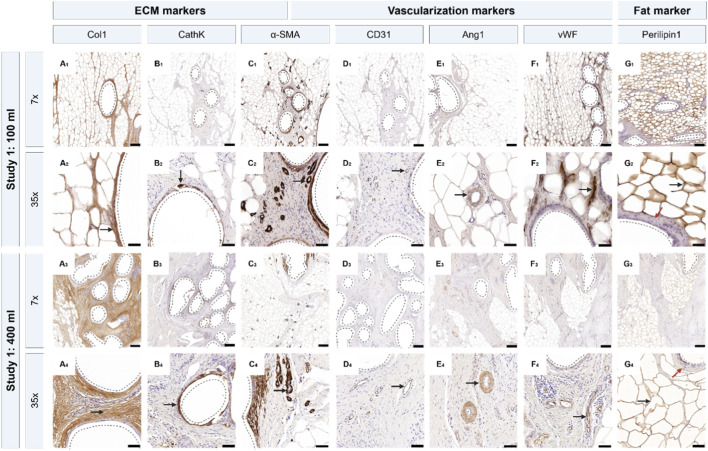
Immunohistochemical visualisation of ECM formation, vascularization, and fat regeneration in 100 ml (study 1) and 400 ml (study 2) volume porous mPCL breast scaffolds (mPCL scaffold only group) 12 months after implantation in a porcine model. The ECM within the connective tissue surrounding the mPCL struts is characterized by the presence of Col-1 **(A**_**1-4**_ **)**. CathK staining was detected in cells at the direct interface with the mPCL scaffold, specifically in macrophages and FBGCs (**B_1–4_**, arrows). Activated myofibroblasts as well as newly formed blood vessels (**C_2,4_**, arrows) are identified by α-SMA staining. Additional vascular markers, including CD31, Ang1, and vWF, demonstrate the formation of a vascular network that permeates the regenerated tissue (**D-F_1–4_**, arrows). The presence of mature white adipocytes is detected by Perilipin1 staining **(G_1–4_)** staining, indicating the regeneration of adipose tissue next to the connective tissue (red arrow) surrounding the mPCL scaffold (black arrow). mPCL struts are delineated with a grey dashed line. Scale bars: 200 µm **(A–G**_**1,3**_ **)** and 50 µm **(A–G**_**2,4**_ **)**.

### Immune response

Central to evaluating immunological processes during tissue regeneration and remodelling within the porous architecture and on the mPCL surface is the assessment of macrophages, vital components of the cellular immune response to foreign biomaterials (Klopfleisch, 2016; Klopfleisch and Jung, 2017; Sussman et al., 2014; Anderson et al., 2008). CD68 is a general macrophage marker that stains both M1 and M2 macrophages and their subtypes, thereby enabling comprehensive assessment of macrophage presence within the regenerated tissue (Klopfleisch, 2016). A distinct staining pattern was observed at the scaffold-tissue interface (Figure 1A_1–4_; Supplementary Figure S1A_1–7_), showing macrophages within the regenerated tissue adjacent to the scaffold struts and to a lesser extent at the contact surface of the mPCL scaffold, similar to patterns observed in prior studies on bone regeneration (Finze et al., 2023). For a more precise phenotypic characterization of macrophages at the mPCL contact site, the expression of iNOS and IRF-5 was examined, commonly described as pro-inflammatory M1 macrophage markers (Murray, 2017). iNOS abundance was specifically detected in the cytosol of macrophages adjacent to the scaffold and within multinucleated foreign body giant cells (FBGC) located at the contact surface of the mPCL scaffold (Figure 1B_1–4_; Supplementary Figure S1B_1–7_). Similarly, IRF-5 positive macrophages exhibited comparable staining and location patterns (Figure 1C_1–4_; Supplementary Figure S1C_1–7_). However, unlike iNOS positive macrophages, IRF-5 labelled macrophages showed higher expression at the scaffold surface within the implant centre of 400 mL scaffolds. Established macrophage markers were used to visualize the more diverse class of M2 macrophages, such as Arg-1, an enzyme that metabolizes arginine to ornithine and urea, thereby inhibiting iNOS by depleting its substrate (Shapouri-Moghaddam et al., 2018; Mosser and Edwards, 2008; Hesse et al., 2001; Lumeng et al., 2007). The results of this staining highlighted Arg-1 positive macrophages predominantly at the tissue transition zones adjacent to the mPCL scaffold, as well as consistently interstitially surrounding the central zone of the implant (Figure 1D_1–4_; Supplementary Figure S1D_1–7_). CD206, also known as mannose receptor (MR) and a well-established M2 macrophage marker in mouse and human, was prominently stained within the regenerated connective tissue within the pore architecture, excluding the direct contact surface with the mPCL scaffold (Figure 1E_1–4_; Supplementary Figure S1E_1–7_). Moreover, these macrophages were regionally distributed around the mPCL strut surface without direct contact, typically enveloping the struts in the second or third cell layer (Figure 1D_2,4_,E_2,4_, arrows; Figure 4) Furthermore, samples were examined for LYVE1 expression (Figure 1F_1–4_; Supplementary Figure S1F_1–7_), originally a marker for endothelial cells of lymphatic vessels, which has recently been associated with an ECM modulating and angiogenesis promoting macrophage subtype (Kieu et al., 2022; Chakarov et al., 2019; Cho et al., 2007). LYVE1 was specifically and consistently detected on macrophages residing in the connective tissue further from the mPCL strut (Figure 1F_2_), as well as, on the surface of the mPCL scaffold, with particularly strong expression in FBGCs (Figure 1F_4_, black arrow). As an endothelial cell marker for lymphatic vessels, LYVE1 staining additionally enabled the visualization of a newly formed lymphatic network within the scaffold which, predominantly located in the ECM-rich connective tissue surrounding the implanted scaffold (Figure 1F_2_, red arrow).

### Extracellular matrix

Collagen type I was detected in the connective tissue surrounding the mPCL struts (Figure 2A_1–4_; Supplementary Figure S2A_1–7_). The collagen fibres formed parallel to the struts' surface but did not establish direct contact with the polymer (Figure 1A_2,4_, arrows). The presence of CathK, a protease predominantly expressed in bone by osteoclasts and a well-established marker for these cells, was specifically localized at macrophages and FBGCs (first layer of cells) at the mPCL scaffold ECM implant-interface (Figure 2B_1–4_, arrows; Supplementary Figure S2B_1–7_). α-SMA, a marker for activated myofibroblasts (Noskovicova et al., 2021), was positively stained within the connective tissue that developed next to the mPCL struts (Figure 2C_1–4_, arrows; Supplementary Figure S2C_1–7_). Investigation of 400 mL scaffolds revealed a gradient of α-SMA staining intensity, with a gradual decrease toward the periphery of the scaffold architecture. Fibrous encapsulation, in the sense of a formation of a distinct acellular layer of parallel-oriented collagen fibres, was not observed. Instead, tissue integration was noted, where ECM-dense connective tissue formed the transition between mPCL scaffold and regenerated adipose tissue within its large pores.

### Vascularization

Projecting infiltration of blood vessels into the scaffold was observed 12 months post-implantation. Numerous vascular structures exhibiting bifurcations were identified within the newly generated tissue (Figures 2C‐F_1–4_, arrows; Supplementary Figure S2C‐F_1–7_). Qualitative analysis of the regenerated tissue within 400 mL scaffolds revealed that the density of these neovascular formations exhibited a gradient, decreasing progressively from the periphery towards the central region of the implant. Notably, these vascular structures were densely concentrated in areas proximal and adjacent to the mPCL scaffold (Figures 2C,D_1–4_; Supplementary Figure S2C,D_1–7_). Staining with α-SMA revealed significant immunolabeling of vascular structures within the regenerated construct (Figure 2C_2,4_; black arrows), even within blood vessels located in the innermost regions of the implant at 12 months (Figure 3). CD31 expression was evident in the newly formed blood vessels progressing from the periphery to the core, with marked staining intensity increasing towards the centre of the implant (Figure 2D_2,4_, arrows). Ang1 immunoreactivity was prominently detected in blood vessels forming adjacent to the mPCL scaffold (Figure 2E_2_, arrow; Supplementary Figure S2E_1–7_) and in vascular structures located further from the scaffold strut (Figure 2E_4_, arrow). The remodelling of the vascular network was further evaluated using vWF staining (Figure 2F_1–4_; Supplementary Figure 2F_1–7_), showing a well vascularized tissue within the scaffold struts. However, it should be noted that in study 2, which investigated the implantation of a 400 mL scaffold, a central zone within the scaffold was observed in a few cases, characterized by the absence of blood vessels. In these instances, none of the vascularization markers mentioned above were positively identified within this zone.

**FIGURE 3:**
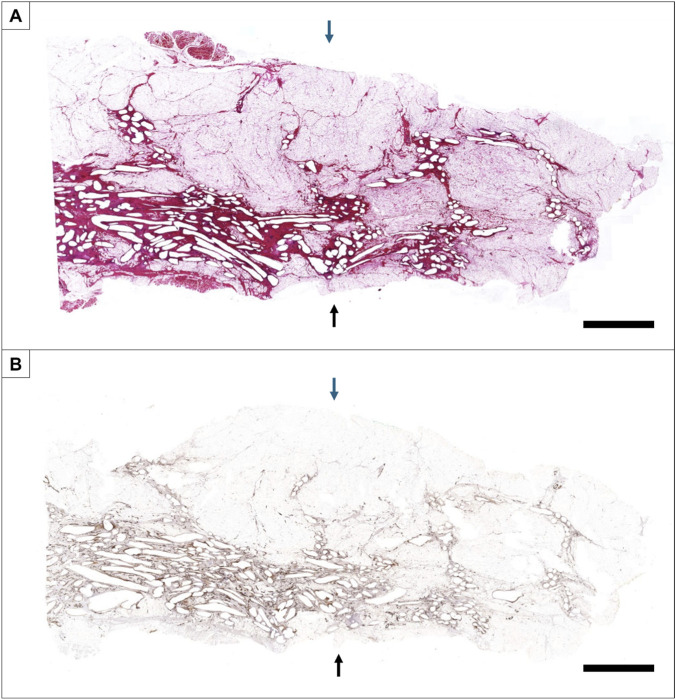
Histological **(A)** and immunohistochemical **(B)** visualization of a cross-section of half of the implant (mPCL scaffold only group, 100 ml), demonstrating extensive and homogeneous tissue integration within the large scaffold pores. Hematoxylin and eosin (H&E) staining **(A)** highlights robust tissue ingrowth originating from both the muscular side (panniculus carnosus muscle; black arrow) and the fascial side with the adjacent adipose tissue (Blue arrow). Prominent vascularization from both the muscle (black arrow) and fascial (blue arrow) compartments is further demonstrated by α-SMA immunostaining **(B)**, which identifies mature blood vessels and myofibroblasts around the scaffold struts. Scale bars: 5000 µm **(A,B)**.

### Fat

Perilipin1 staining confirmed the presence of vital adipocytes through specific labelling of their cell membranes (Hansen et al., 2017; Eto et al., 2012). Particularly high staining intensity was observed in the outermost zone of the implant, where positively marked adipose cells infiltrated towards the scaffold centre and organized themselves between the mPCL struts (Figure 2G_2,4_, black arrows; Supplementary Figure 2G_1–7_). The presence of adipocytes in direct proximity to the implanted mPCL scaffold was rare. Instead, these cells formed near the ECM-rich connective tissue adjacent to the mPCL scaffold (Figure 2G_2,4_; red arrows; Supplementary Figure 2G_1–7_). The formation of adipose cells was particularly prominent in regions where the distance between mPCL struts was ensured, respectively, within the scaffold’s 5–8 mm pores (400 mL). In contrast, areas where mPCL struts were positioned closer together, limiting the scaffold’s ability to create spaces conducive to adipogenesis, exhibited reduced fat tissue regeneration.

### mPCL integration and soft tissue regeneration in the context of SGBR

The implantation of a porous biodegradable mPCL scaffold elicits a stepwise cascade of immune responses, as characterized by immunohistochemistry of cellular phenotypes in the peri-scaffold tissue, as illustrated in Figure 4A. Cells directly contacting the mPCL scaffold include macrophages and foreign body giant cells (FBGCs), marked by iNOS, IRF-5, LYVE1, and CathK expression (Figures 4A,B). Adjacent to this interface is a zone of alternatively activated macrophages expressing Arg-1 and CD206, which are also dispersed throughout the fibrous connective tissue surrounding the scaffold struts (Figures 4A,B). CD68-positive cells are found both adherent to the mPCL surface and distributed within the surrounding matrix. The fibrous connective tissue is characterized by α-SMA positive myofibroblasts and dense collagen type I deposition. Endothelial cells forming neovessels within the peri-scaffold sheath are identified by co-expression of CD31, Ang1, vWF, and α-SMA (Figures 4A,B). At a greater distance from the scaffold, white adipocytes, marked by Perilipin1, become increasingly prominent (Figures 4A,B). More importantly, formation of adipose tissue is particularly pronounced in regions where spacing between mPCL struts is maintained within farther distance (Figure 4C).

**FIGURE 4:**
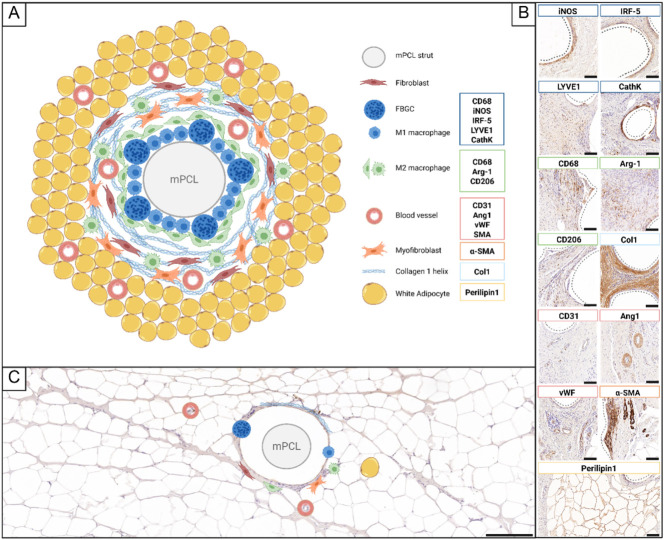
Characteristic spatial distribution of immunolabelled cells and ECM in relation to mPCL scaffold location within regenerated tissue. **(A)** Schematic representation of the regional distribution of specific cell types within the regenerated tissue around the mPCL scaffold struts, as detected by antibodies used in this porcine model. The first layer of cells directly in contact with the mPCL scaffold comprises FBGCs and macrophages, characterized by the expression of iNOS, IRF-5, LYVE1, and CathK. Adjacent to this layer, a further zone of macrophages expressing Arg-1 and CD206 is present, which are also disseminated within the fibrous connective tissue surrounding the mPCL struts. CD68 positive cells can be found both directly adhering to the mPCL surface and within the surrounding connective tissue. This connective tissue is marked by the presence of myofibroblasts expressing α-SMA and the deposition of collagen type 1. Endothelial cells in newly formed blood vessels, which are densely located in the peri-scaffold connective tissue sheath, express CD31, Ang1, vWF, and α-SMA. At a greater distance from the mPCL scaffold, the formation of white adipocytes expressing Perilipin1 is characteristic. **(B)** IHC images (mPCL scaffold only group) were generated using the antibody panel to label the structures illustrated in A specifically. mPCL struts are delineated with a grey dashed line. **(C)** Haematoxylin counterstaining of tissue section (study 1), demonstrating the characteristic spatial arrangement of distinct cell types illustrated in **(A)**. Scale bar: 50 µm **(B)** and 200 µm **(C)**.

## Discussion

Before new TE&RM technologies are applied to humans for the first time, it is essential to study them in experimental models that closely mimic human physiological and anatomical conditions. This ensures that the potential risks and benefits are thoroughly understood before the planning and execution of the first-in-human study (Mukherjee et al., 2022). Using rodent models to study breast tissue engineering provides valuable proof-of-concept data; however, their clinical relevance is limited by the inability to replicate the anatomical and physiological volumes required for human breast reconstruction. The porcine model offers a promising alternative because soft-tissue architecture in humans and pigs is similar, yielding clinically translatable regenerated volumes and enabling extensive IHC analysis that is often not feasible in clinical trials. IHC protocols were validated in a preclinical porcine model as an investigative tool to evaluate ECM formation and immune cell responses in SGBR over 12 months.

The immune system’s reactions to the surgical implantation of a foreign body at all times initiate cascades of both adaptive and innate immune responses, which integrate fundamental concepts of wound healing and FBR, ideally leading to successful tissue regeneration and homeostasis (Klopfleisch and Jung, 2017; Nepon et al., 2024). These responses are influenced by various properties of the implant, such as its porosity, pore size and architecture, surface roughness, charge and hydrophilicity, and mechanical properties (Capuani et al., 2022; Doloff et al., 2021). Other factors that influence outcomes include the animal model, surgical technique, implantation site, and any additional therapy. If the scaffold is combined with an autologous (e.g., AFG and PRP) or allogeneic graft, additional factors in the host immune response need to be studied (Klopfleisch and Jung, 2017; Anderson et al., 2008; Sadtler et al., 2019).

After creating a pocket for breast scaffold placement, blood clot formation is initiated by immediate protein adhesion to the scaffold surface. Provisional fibrin matrix formation and the onset of an acute inflammation follow before transitioning into a more chronic inflammatory response, which ultimately culminates in tissue regeneration and remodelling (Luttikhuizen et al., 2006; Chandorkar et al., 2019). Although these stages share similarities with natural wound healing and FBR, tissue regeneration and FBR progression should not be viewed as two separate phenomena in the context of SGBR. It should be evaluated as a set of regionally and temporally interconnected processes that influence one another, ultimately determining the regenerated outcome (Finze et al., 2023).

There is a critical need to characterize both the cellular and ECM responses of the surrounding tissue to the implantation stimulus, in order to elucidate and better understand the biological behaviour of periprosthetic tissue. Across various medical procedures, these responses are anticipated and play a critical role in determining an intervention’s outcome by influencing tissue integration and regeneration (Pluvy et al., 2025; Jones et al., 2025). This involves conducting comprehensive IHC analyses to evaluate different cell types and examine the composition and structure of the ECM, which display diverse differentiation and expression patterns, as these factors contribute to the complex environment in which inflammatory and regenerative processes occur. It is equally essential to clarify the relationship between the expression patterns of various cell types and ECM components and the clinical relevance of fibrosis formation in the context of SGBR, which differs markedly from that observed when assessing fibrosis in the clinical gold standard of silicone implants.

Rather than conducting comparative analysis across various implant designs, experimental groups, and temporal endpoints, this discourse will concentrate on fundamental aspects and inter-category observations regarding the immune response, ECM formation, vascularization, and fat formation elicited by the mPCL scaffold.

### Immune response

Macrophages, derived from circulating monocytes, are vital components of the immune system and play a critical role in the body’s response to the implantation of foreign material, migrating to the implantation site via the bloodstream (Klopfleisch and Jung, 2017). Their behaviour and interactions within the body are a key focus of research in understanding immunological responses to foreign body implantation, making them particularly significant in the context of the SGBR domain, which encompasses the study of surgical grafts, biocompatibility, and tissue regeneration (Klopfleisch, 2016; Klopfleisch and Jung, 2017; Liu et al., 2025; Ward, 2008). As a profoundly heterogeneous cell type, macrophages are typically classified into M1 and M2 phenotypes based on numerous *in vitro* differentiation studies, in which M1 activation is induced by interferon-gamma (IFN-γ) and Lipopolysaccharide (LPS), and M2 activation is induced by Interleukin-4 (IL-4) and Interleukin-13 (IL-13) (Mantovani et al., 2004). These correspond to an inflammatory and a pro-regenerative phenotype, respectively. M2 macrophages encompass multiple subtypes with distinct protein expression patterns and associated functions (Klopfleisch, 2016). It is acknowledged that this dichotomy of phenotypes likely oversimplifies the complex *in vivo* function, as macrophages exhibit a spectrum of activation and expression patterns, thereby elucidating the concept of macrophage plasticity (Martin and García, 2021; Martinez and Gordon, 2014). However, this discussion will adhere to the classification of M1 and M2 macrophage subtypes to facilitate the illustration of inflammatory and regenerative processes.

As decisive immune cells in the innate immune response, macrophages play a central role in responding to foreign material by actively altering the implant microenvironment through their various activation states (Klopfleisch, 2016). In the studies conducted, the surface of the mPCL scaffold exhibited positive staining for the nonspecific macrophage marker CD68, indicating regeneration and tissue remodelling. In the case of large-volume tissue formation targeted by large scaffolds, this process can take an extended period before reaching a state of physiological homeostasis. The physicochemical properties of the mPCL scaffold surface enhanced protein adsorption from locally present wound fluids generated during implantation surgery, thereby facilitating macrophage adhesion. The positive staining pattern for CD68 (marker for M1 and M2 macrophages) on the mPCL scaffold’s surface indicates cell-biomaterial interaction in all cases.

Although it has been proposed that a transition from M1 to M2 phenotype, which fosters a favourable wound-healing environment, can occur, both M1 and M2 macrophage subtypes were present simultaneously across all study groups. Instead of observing a phenotypic switch that depends on a distinct wound healing or tissue regeneration stage with one predominant macrophage subtype, various macrophage subtypes were present simultaneously across all study groups, revealing a characteristic protein expression pattern associated with their spatial distribution relative to the scaffold’s architecture.

The expression of IRF-5 in M1 macrophages, along with the concurrent upregulation of inflammatory gene transcription, has been linked to increased secretion of inflammatory cytokines, including tumor necrosis factor alpha (TNF-α), Interleukin-6 (IL-6), Interleukin-12 (IL-12), and Interleukin-23 (IL-23). This elevated release promotes macrophage polarization toward an inflammatory phenotype and enhances T-lymphocyte activation, leading to the development of T-helper cell type 1 (TH1) and T-helper cell type 17 (TH17) phenotypes (Ouyang et al., 2007; Chistiakov et al., 2018; Krausgruber et al., 2011). These processes are associated with enhanced microbicidal and phagocytic capacities in macrophages, thereby aiming to protect the organism from pathogens (Krausgruber et al., 2011). The accumulation of IRF-5 and iNOS-positive M1 macrophages at the mPCL surface, as an attempt to recognize and phagocytize the foreign mPCL struts, highlights the macrophage and T-cell-driven inflammatory microenvironment directly surrounding the mPCL. Yet, due to the inability to internalize the mPCL struts in its entirety, FBGCs (fused macrophages), adhered to the mPCL surface to accelerate its degradation, thereby facilitating its incorporation and elimination from the body (Anderson et al., 2008). Concomitantly, increased iNOS expression in this subset of macrophages and FBGCs reinforces their efforts to incorporate and eliminate the scaffold, as if it were a pathogen, through the generation of reactive oxygen species (ROS). *In vitro* studies have demonstrated that an acidic environment, presumably generated at the scaffold interface during this immune response, can expedite degradation of the implanted mPCL scaffold by enhancing bulk degradation (Bartnikowski et al., 2019).

Notably, our experiments also showed that the most intense CathK immunolabeling was observed in IRF-5- and iNOS-positive macrophages, specifically at FBGCs in direct contact with the mPCL scaffold, suggesting that CathK may serve as a marker for FBGCs in a porcine model. Prior studies have linked CathK to activated macrophages and multinucleated giant cells (MGC) (subtype of FBGCs), supporting its role as a marker for macrophage differentiation (Bühling et al., 2001). The specific presence of CathK labelled cells near the scaffold interface, in a region dominated by ECM and connective tissue, suggests that CathK-positive macrophages are unlikely to exhibit anti-fibrotic properties in the context of SGBR in a porcine model (Adhyatmika et al., 2015). Instead, they appear to be associated with ECM (collagen type I) remodelling, consistent with previous findings on their role in osteoclastogenesis (Drake et al., 1996; Inaoka et al., 1995).

Next to the layer of M1 macrophages on the outer surface of the scaffold, a distinct expression of proteins associated with M2 macrophages, such as Arg-1 and CD206 was observed. Due to their heterogeneity, these pro-regenerative considered macrophages are more challenging to categorize into a single subtype; thus, the pro-regenerative M2 subtype is further subdivided into M2a, M2b, M2c, and M2d (Klopfleisch, 2016; Witherel et al., 2019). Each designated subtype is believed to have a specific polarization stimulus, as well as a specific pattern of expressed genes, secreted effector molecules, and respective functions (Wang et al., 2014). Arg-1, an enzyme that competitively inhibits iNOS expression, is reported to be associated with pro-healing and pro-fibrotic outcomes (Klopfleisch, 2016; Murray, 2017; Rath et al., 2014). In a mouse model, Pesce et al. demonstrated that macrophages expressing Arg-1 were responsible for suppressing fibrosis development and were therefore considered anti-fibrotic in this context (Pesce et al., 2009). In the investigated porcine model, the distribution of Arg-1-expressing macrophages displayed a distinct arrangement in the vicinity of the mPCL struts. Unlike the previously mentioned iNOS and IRF-5 marked M1 macrophages, Arg-1 positive M2 macrophages did not exhibit direct attachment to the scaffold surface, nor could staining of FBGCs thoroughly be observed. Instead, Arg-1 macrophages were located closer to the regenerating tissue in between the scaffold struts. This tissue, characterized mainly by collagen type I as an extracellular component, was interspersed with Arg-1-positive macrophages, suggesting the influence of their previously described anti pro-fibrotic and tissue-repairing properties, via the generation of ornithine and ultimately proline and polyamines, on its surrounding tissue (Hesse et al., 2001; Kreider et al., 2007). Notably, cytokines such as Transforming Growth Factor-β (TGF-β), Insulin-like Growth Factor 1 (IGF-1), and Platelet-Derived Growth Factor (PDGF), secreted by Arg-1-positive macrophages, have been described to enhance ECM deposition and influence fibroblasts’ behaviour, leading to ECM remodelling through the modulation of matrix metalloproteinases expression (Franchini and Ottaviani, 2000; Wynn, 2008). These processes are crucial for tissue repair and for preventing fibrosis.

Sussman et al. have proposed that the cellular response and the degree of collagen deposition during the FBR in a subcutaneous mouse model are influenced by the pore size of the implanted device (Sussman et al., 2014). Specifically, scaffolds with 160 μm pores exhibited greater collagen-rich cellular infiltration compared to non-porous scaffolds, which were surrounded by a significantly thicker foreign body capsule, as confirmed histologically. In contrast, scaffolds with 34 μm pores exhibited a high presence of M1 macrophages within the pores, while there was a predominance of M2 macrophages outside the pores. These scaffolds demonstrated less collagen deposition but exhibited the highest formation of blood vessels, as indicated by CD31 staining (Sussman et al., 2014). These findings suggest that there is a pore size that supports tissue regeneration and modulates the FBR, particularly through macrophage activity. Our results support this hypothesis. In the studies conducted, a trend was observed in which cells expressing M1 macrophage markers colonized the contact interface of the macro-porous mPCL scaffolds. Conversely, cells expressing M2 markers tended to accumulate in adjacent areas. However, determining an optimal pore size that directs the FBR toward a desired outcome by modulating the immune response is complex. It depends on several parameters, such as the animal model used (species, implantation site, and surgical technique). A single measurement cannot conclusively establish it at a specific time point after explantation. This complexity underscores the need for comprehensive investigation in future studies.

### Extracellular matrix (ECM)

The deposition of collagen-containing ECM in association with implants is controlled by the FBR, which significantly impedes the long-term success of many implants, leading to implant failure and requiring reoperation (Coroneos et al., 2019). Fibrosis occurs when ECM production exceeds ECM degradation, resulting in an imbalance that favours ECM accumulation, macrophage activation, and subsequent differentiation of fibroblasts into myofibroblasts, producing a collagen-rich ECM that culminates in fibrotic capsule formation around the implant (Wynn, 2008; Bryers et al., 2012; Safran et al., 2021).

Silicone implants often induce an immune response with subsequent tissue formation that conforms closely to the implant’s surface, resulting in capsule formation that encloses the implant as a whole (Larsen et al., 2021). This process is further exacerbated by myofibroblast-mediated contractility of the formed fibrotic capsule, which can clinically manifest as capsular contractures (Larsen et al., 2021; Hwang et al., 2010). This transition involves the incorporation of α-SMA-positive stress fibres, imparting contractility to the myofibroblasts (Noskovicova et al., 2021; Mariani et al., 2019). Compared with silicone implants, our studies indicate that a similar process occurs with mPCL scaffolds. Our results showed an accumulation of α-SMA-positive cells and increased collagen type I deposition adjacent to the mPCL struts, particularly in the vicinity of Arg-1- and CD206-positive macrophages, presumably due to their pro-fibrotic capacities (Murray, 2017). This peri-implant ECM envelops the mPCL scaffold struts like a sheath, providing support and stability to the regenerated tissue (Figure 5). In contrast, the scaffold itself provides sustained mechanical support that resists myofibroblast contractility and maintains volume and shape.

**FIGURE 5:**
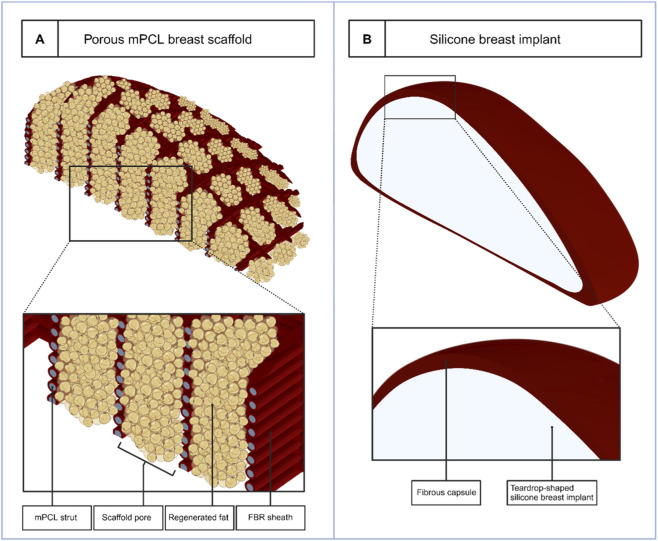
Schematic comparison of tissue response and FBR in porous 100 mL mPCL scaffolds **(A)** and expected FBR in silicone breast implants **(B)**. **(A)** Schematic representation of regenerated tissue in a cross-sectioned mPCL scaffold. The architectural design of the additively manufactured porous mPCL scaffold dictates the course of the FBR (red), which occurs along the surface of individual mPCL struts (blue). The scaffold’s structural stability facilitates adipose tissue regeneration within its large pores, where adipocytes are integrated into ECM-dominated connective tissue, without displaying direct contact with the scaffold. **(B)** Schematic representation for the illustration of the fibrotic capsule (red) formation typically induced by the FBR around a cross-sectioned silicone implant (light blue). The FBR proceeds along the implant’s surface, entirely encapsulating it. In contrast, within the mPCL scaffold, the FBR follows the individual mPCL struts from which the scaffold is constructed, allowing for adipose tissue regeneration within the large pores without encapsulating the scaffold in its entirety. This figure is intended for illustrative purposes only to depict potential differences in FBR related to implant design and does not represent a direct experimental comparison, as solid implants were not included in the study design. mPCL = medical-grade Polycaprolactone; FBR = foreign body response.

Doloff et al. have demonstrated that the surface properties of polydimethylsiloxane (PDMS) silicone breast implants, particularly their texturing with a roughness of 0–90 μm, can significantly influence the extent of the FBR in rabbit and mouse studies (Doloff et al., 2021). Specifically, silicone breast implants with a 4 µm texture induced the mildest histologically and IHC-confirmed FBR (Doloff et al., 2021). Compared with porous mPCL scaffolds with porosity exceeding 90%, the inflammatory response elicited correlated with the architecture of their additively manufactured struts. This response led to the formation of a connective tissue layer that did not uniformly encapsulate the scaffold, unlike anatomically shaped PDMS silicone implants. Instead, it followed the contour of the struts throughout the scaffold’s architecture. These two distinct processes are schematically represented in Figure 5. Briefly, cells initially attach to the scaffold surface and deposit ECM aligned with their body axes, a process influenced by mechanical cues that induce cell stretching. Through this mechanism, cells actively organize and build the ECM, and therefore collagen deposition within the scaffold porous architecture. In turn, the porous architecture of the mPCL scaffold, fundamentally influences the arrangement of the FBR. Rather than forming a continuous fibrous capsule at the implant periphery, as commonly described for solid implants, the host response follows the surface of individual struts and extends throughout the interconnected pore network into the interior of the scaffold. This results in a decentralized, strut-associated fibrous response, rather than a single circumferential capsule (Figure 5). In this SGBR context, ECM-dominated connective tissue serves as a supportive network for the migration, differentiation, and proliferation of adipocyte cells, without them coming into direct contact with the scaffold itself (Figure 5A). The scaffold’s structural stability and large pores then provide a free surface to support adipose tissue regeneration. In contrast, silicone implants elicit a distinct FBR that progresses along the implant surface, resulting in complete fibrous encapsulation (Figure 5B). However, unlike mPCL scaffolds, silicone implants are not designed to promote adipose tissue regeneration, highlighting a significant functional distinction between the two materials and designs.

The extent of fibrous encapsulation is typically regarded as a negative outcome in silicone implant-based breast reconstruction, clinically assessed using standardized scoring systems, such as the Baker Classification for capsular contracture (Spear and Baker, 1995). Conversely, our findings suggest that in highly porous breast scaffolds, macrophage-driven ECM deposition plays a regenerative role by establishing a fibrous template network that promotes adipogenesis and angiogenesis while providing structural stability to the developing tissue analogous to Cooper’s ligaments. This behaviour reinforces the broader concept of SGBR. In this context, the mPCL scaffold does not passively receive a FBR but actively guides tissue formation within its internal geometry, promoting distributed remodeling rather than peripheral isolation. It is plausible that spatial variations in curvature and orientation across interconnected struts induce gradual shifts in cell alignment, leading to layered or rotated collagen deposition patterns. It has been shown *in vitro* that cells preferentially align along convex surfaces, adopting cytoskeletal organization dictated by local geometry (Schamberger et al., 2024). In turn, collagen deposition follows this cellular alignment, as cells secrete ECM parallel to their actin cytoskeleton. This results in anisotropic collagen organization along scaffold struts, rather than the isotropic deposition typical of dense capsule formation.

Although aliphatic polyesters such as mPCL are known to undergo hydrolytic degradation through either surface or bulk erosion mechanisms, the formation of fibrous tissue around the scaffold struts does not impede scaffold degradation (Bartnikowski et al., 2019). Instead, degradation continues locally, mediated by macrophages. Increased iNOS expression in this subset of macrophages and FBGCs supports their capacity to incorporate and eliminate the scaffold through the local generation of ROS, which in turn facilitate material breakdown and elimination. As the scaffold gradually degrades, the regenerated tissue undergoes continuous rounds of dynamic remodelling, which should be considered when interpreting histological and IHC results, as the observed tissue composition may not reflect its final regenerated state.

Therefore, it is essential to evaluate fibrosis in the context of biodegradable, highly porous scaffolds differently than in solid silicone implants, as the processes in each context carry distinct implications for tissue integration and clinical outcomes. This discrepancy is underscored by recent findings that periprosthetic tissue in silicone implants expresses immune markers commonly observed in autoimmune disorders, raising concerns about the immunological safety of such devices (Pluvy et al., 2025).

### Vascularization

Ensuring an ample blood supply to tissue-engineered constructs is essential for successful tissue regeneration. However, the challenge of achieving neovascularization remains a significant hurdle in preclinical research and clinical translation (Lovett et al., 2009). Notably, both M1 and M2 macrophages are pivotal in facilitating the formation of new blood vessels, each playing a crucial role at different stages of the regeneration and remodelling phases (Martin and García, 2021). Endothelial cell sprouting is associated with pro-inflammatory M1 macrophages, which secrete vascular endothelial growth factor (VEGF), promoting the initial formation of new blood vessels. Conversely, M2 macrophages are thought to be more involved in blood vessel maturation (Spiller et al., 2014). Staining procedures used to visualize these processes are consistent with these theories. A high density of CD31, Ang1, vWF, and α-SMA positive cells was observed within the centre of the scaffold pores and near macrophage populations predominantly arranged around the scaffold interface, yet not directly at the scaffold strut surface. Markers associated with early angiogenesis, such as Ang1, and those linked to later stages of vessel maturation, such as vWF and α-SMA, were ubiquitously expressed without regional intensity differences relative to the scaffold architecture (Abdel-Malak et al., 2009; Nillesen et al., 2007). However, the increasing positive signal of the vascular endothelial marker CD31 observed towards the inner centre of the scaffold indicates the relatively younger phenotype of these blood vessels, suggesting that the further peripheral located blood vessels are already in a later stage of angiogenesis (Sass et al., 2017). This phenomenon elucidates the ingrowth of blood vessels from the surrounding tissue and the subsequent progressive infiltration of these newly formed vessels into more profound regions within the scaffold over time. From this, one can deduce that the immunological response to mPCL scaffold implantation in the porcine model can create a microenvironment conducive to neoangiogenesis and subsequent blood vessel maturation, ensuring adequate blood supply within the scaffold architecture.

### Fat

SGBR is not designed to precisely replicate all aspects of the anatomical breast structure, such as the mammary glands and lactiferous ducts. Since a significant proportion (80%–90%) of the female breast comprises stromal tissue dominated by adipocytes, mechanically suspended by Cooper ligaments, a combination of white adipocytes and fibro-ligamentous connective tissue is considered optimal for providing both a natural feel and mechanical stability to achieve the desired breast shape (Patrick, 2004). The specific detection of viable adipocytes, indicated by the presence of Perilipin1 within the pores of the scaffold, suggests the formation of functional, mature adipocytes. Given the high microenvironmental demands of adipocytes, ensuring adequate nutrient and waste transport is crucial for their sustained functionality (Eto et al., 2012; Pope et al., 2016). The IHC results presented indicate that these conditions can be met by implanting highly porous mPCL breast scaffolds. Yet, spatial differences were observed in tissue composition: regenerated adipose tissue was predominantly found within the larger scaffold pores, whereas fibrous connective tissue was located closer to the scaffold surface and was characterized by immune-cell-rich and ECM-driven features. Thus, in alignment with the conducted IHC ECM investigations, it can be concluded that pore size affects the quality and generation of extracellular tissue components, particularly collagen type 1 deposition, and profoundly orchestrates cellular constituents to form new tissue. Importantly, preadipocyte migration, proliferation and differentiation into the scaffold pores must be conveyed by targeted chemotaxis, as cellular differentiation towards the adipocytic cell lineage is not found as part of the natural healing process (Fitridge and Thompson, 2011). In this context, TGF-β and fibronectin have been identified as key moderators for preadipocyte migration (Mao et al., 2022). Moreover, the IHC results demonstrate adipose tissue formation predominantly at the scaffolds’ periphery, reaching towards the scaffolds’ centre mainly through column-like large-diameter (5–8 mm) pore-channels in contrast to smaller pores, where fewer adipocytes were found to expand towards the scaffold’s core. These distinct findings indicate that, despite the benefits of structural support in tissue remodelling, a collagen-rich ECM, depending on its structural density, can also function as a physical barrier for adipocytic differentiation and migration (Jääskeläinen et al., 2023).

## Limitations and future directions

The primary aim of this work was a qualitative, exploratory evaluation of histological patterns in representative samples rather than a comparative or quantitative analysis across groups. Direct comparisons cannot be made for the present study as the mPCL scaffolds used in Studies 1 and 2 differ in geometry, overall size, and adjunctive treatments, thereby limiting direct comparability. Notably, the observed staining characteristics and spatial distributions were consistent across the analyzed samples, supporting the robustness of these qualitative observations despite the absence of formal quantification. Quantification of the data (Study 1) has been previously reported by Cheng et al. (Cheng et al., 2021; Cheng et al., 2024). Briefly, AFG and PRP were incorporated into the original experimental design based on their well-established biological rationale. In particular, PRP contains a broad range of growth factors, including platelet-derived growth factor, fibroblast growth factor, vascular endothelial growth factor, epidermal growth factor, insulin-like growth factor-1, and transforming growth factor-β, that are known to support early wound healing, angiogenesis, and extracellular matrix formation. The absence of sustained long-term effects of these adjunctive strategies, as previously reported by Chhaya et al. and Cheng et al., reflects the transient nature of these early biological signals rather than a lack of biological relevance (Chhaya et al., 2016; Cheng et al., 2021; Cheng et al., 2024). Indeed, both AFG and PRP demonstrate strong adipogenic and regenerative potential during the early phases of healing, which provides the rationale for their continued investigation and integration within evolving SGBR concepts. PRP and AFG are therefore expected to influence early inflammatory and regenerative phases but is not anticipated to override long-term scaffold-driven remodelling. Consequently, the lack of long-term differences between treatment groups, as previously reported, is biologically meaningful and reinforces the concept that scaffold architecture is the primary determinant of late-stage tissue organization and maturation. Moreover, adipose tissue undergoes multiple cycles of remodelling throughout the wound-healing process, making it impossible to histologically delineate the originally applied PRP or AFG at late time points. For this reason, rather than attributing late-stage tissue features to transient early interventions (PRP and AFG), the present study focuses on a detailed immunohistochemical characterization of the host immune response following long-term scaffold implantation. This approach provides mechanistic insight into scaffold-guided tissue regeneration/remodelling and directly strengthens the scientific narrative of the current IHC analysis of our manuscript.

Currently, there is a notable lack of adequate characterization of soft-tissue regeneration and the immunological processes associated with SGBR in large-animal models. This absence is particularly relevant from a clinical perspective, particularly for the implantation of large-volume scaffolds that can reach up to 400 mL. The porcine model is widely used in biomaterial research due to similarities to humans in skin architecture, wound healing characteristics, and aspects of the immune response, making it a valuable platform for studying the regeneration of large soft-tissue volumes, such as the 100 mL and 400 mL scaffolds investigated herein. The present study aimed to contribute to this gap by providing a detailed, descriptive analysis of the local immune response and tissue composition at a clinically relevant large animal model and time point.

At the same time, important limitations must be acknowledged. These include species-specific differences in immune cell phenotypes, macrophage marker expression, cytokine profiles, and the kinetics of the inflammatory response, as well as potential variability related to the implantation site (Comley and Fleck, 2010; Griffin et al., 2025). In addition, although porcine soft-tissue anatomy closely resembles that of humans, differences in overall healing capacity and physiological parameters, such as higher core body temperature, may influence tissue regeneration and biomaterial integration.

Although in this study we conducted IHC staining using serial sections, and the consistent spatial localization of specific protein markers relative to scaffold structures was reproducible across adjacent sections, supporting a descriptive interpretation of tissue-level spatial organization without implying true cellular co-expression, cellular distribution slightly changes with tissue depth. Definitive statements on co-localization or marker co-expression would be required to confirm co-expression of protein markers, specifically, co-localization of M1 and M2 macrophages like Arg-1/iNOS and or Col1/CD68 to further refine the observations made herein.

Speculations regarding the progression of the FBR following complete degradation of the mPCL scaffold, particularly with respect to the activation patterns of immune cells previously associated with the scaffold struts, still lack in the literature. The prolonged degradation profile of mPCL would require longer studies time points, over periods of 5–6 years, which would require substantial logistical and financial resources, and remains to be demonstrated in future studies.

## Materials

Ethical approval was granted by the Queensland University of Technology (QUT) Animal Ethics Committee (UAEC) (Approval Numbers: 1600000282, 2908). All animal surgeries were conducted at the QUT Medical Engineering Research Facility (MERF) in accordance with the guidelines outlined in the Australian Code for the Care and Use of Animals for Scientific Purposes.

### Study groups

The IHC stainings conducted for this study originated from two large preclinical porcine model studies, with the first study (study 1) involving the implantation of five scaffolds per pig in 11 pigs (n = 55), and a subsequent pilot study (study 2) involving two pigs receiving four implants (n = 8) (Cheng et al., 2021; Cheng et al., 2024). Study 1 utilized additively manufactured scaffolds with a volume of 100 mL, while study 2 investigated commercially acquired scaffolds with a volume of 400 mL, sourced from BellaSeno (Leipzig, Germany) (Cheng et al., 2021; Cheng et al., 2024). All implanted scaffolds were composed of 100% mPCL, with a strut diameter of 350 μm, an overall porosity of 90%, and 100% pore connectivity (Supplementary Figure S3). The scaffold’s architectural design differed between the two studies, as well as the pore size, with 8 mm in study 1 and 5–8 mm in study 2. The scaffolds were left pristine or loaded with AFG or PRP, administered at implantation or at two (study 2) or four (study 1) weeks post-implantation, and injected into the scaffolds via fan-configured channels (Supplementary Table S1). Detailed descriptions of the scaffold design, including its inherent physicochemical properties, as well as specifics of experimental groups and surgical techniques are provided elsewhere (Cheng et al., 2021; Cheng et al., 2024; Mohseni et al., 2019). For clarity, a brief description of the material and methods, as well as an image outlining the main outcomes of our previous work is provided in the supplementary material (Supplementary Figure S4) (Cheng et al., 2021; Cheng et al., 2024). Analysis of scaffolds in study 1 comprised one scaffold per treatment group defined by Cheng et al., while all implants of study 2 were included in the IHC analysis. For each implant under investigation, two distinct tissue sections from different regions within the implant were examined per conducted antibody staining (Supplementary Figure S5). More detailed immunohistochemical visualization of the individual treatment groups from both studies is provided in the supplementary material, including analyses of the immune response (Supplementary Figure S1) as well as ECM formation, vascularization, and adipocyte regeneration (Supplementary Figure S2).

### Surgical protocols

All experiments were conducted on adult female immunocompetent Australian minipigs (*Sus scrofa* domestica), weighing 90–120 kg. The surgical procedures for implantation have previously been described (Cheng et al., 2021). Briefly, longitudinal incisions were placed approximately 20 cm lateral to the nipple row with a length of 10 cm (study 1) and 15 cm (study 2). Dissection was performed through the subcutaneous adipose tissue and panniculus carnosus muscle and fascia, and pockets were formed towards the lateral aspects of the pigs for flank implantation. The incisions were made separately to prevent communication between pockets. Between the panniculus carnosus muscle and deep fascia, a pocket matching the scaffold size was created and prepared to allow tension-free adaptation of the wound edges. After scaffold insertion, the pockets were closed with multilayer sutures of absorbable material and surgical staples. Postoperative care included wound inspection, dressing changes, analgesia, and temporary application of customized protective jackets. AFG and PRP were administered either at the time of implantation or at 2 or 4 weeks later, using a Body-Jet Eco (Human Med, Schwerin, Germany) liposuction system and the Angel System (Arthrex, Naples, FL), respectively. Briefly, 100 mL of autologous adipose tissue was harvested from the abdominal region of the pigs for AFG. Subsequently, 50 mL of processed lipoaspirate was injected into the scaffold pores in a fan-shaped distribution pattern using a 12-gauge needle- Scaffolds allocated to the PRP group received 50 mL pf PRP, which had been pre-activated with 10% calcium chloride to induce gel formation, thereby increasing viscosity and enabling more controlled and spatially precise injection into the scaffold architecture. Humane killing was performed 12 months post-implantation through intravenous administration of Lethabarb® at a dosage of 100 mg/kg. Post humane killing, the specimens were explanted, suture marked for orientation, and subjected to magnetic resonance imaging (MRI), computed tomography (CT), and biomechanical testing. Following the completion of non-failure biomechanical testing, scaffolds were labelled with tissue dye for orientation and sectioned into 1–2 cm blocks to facilitate fixation in 4% paraformaldehyde (PFA) (Sigma Aldrich, St. Louis, United States) at a ratio of 15 parts fixative to 1 part tissue, maintained for periods of five (study 1) and 7 days (study 2). Post-fixation, specimens were stored in 70% v/v ethanol at 4 °C pending further analysis. Effective coordination between the specimen collection team and the histology specialist ensured strict adherence to established collection protocols (Finze et al., 2023). Minimizing the time from circulatory arrest of the animal to fixation of the regenerated construct were critical for mitigating the degradation of key biomarkers, particularly proteins prone to autolysis, commonly found in metabolism-sensitive cells such as adipocytes. Detailed information about the methodology used in the study by Cheng et al. can be found in the supplementary information on methodology of Supplementary Figure S4 (Cheng et al., 2021; Cheng et al., 2024).

### Immunohistochemical analysis

The tissue response to the implantation of mPCL scaffolds, focusing on inflammatory reaction, ECM formation, vascularization, and adipose tissue regeneration, was systematically evaluated using paraffin-embedded samples. Fixed tissue samples were serially dehydrated and paraffin-embedded using an automated Excelsior ES tissue processor for 16 h (Excelsior ES, Thermo Scientific, Franklin, MA, United States). The processed samples were then embedded in molten paraffin wax at 60 °C into supa-mega cassettes (Thermo Fisher Scientific, United Kingdom). 5 μm sections were cut using a standard rotary microtome (Leica Biosystems, Nussloch, Germany) with disposable blades (Feather, Osaka, Japan) and an extra-large clamp to fit the supa-mega cassettes, collected on poly-lysine slides (Polysine Adhesion Microscope Slides, Epredia, United States), and oven-dried at 60 °C for 24 h. Slides for IHC staining were prepared following established protocols (Finze et al., 2023; Sparks et al., 2020). Antigen retrieval methods included the use of Proteinase K, as well as heat-induced antigen retrieval using 10 mM Tris-Ethylenediaminetetraacetic acid (EDTA) buffer (in 1.21 g TRIS add 0.37 g EDTA; add 1 L H2O and 0.5 mL Tween 20, pH 9.0) and 10 mM Tris-Citrate buffer (1.45 g Tri-sodium Citrate Dehydrate in 500 mL distilled water; pH to 6.0; add 250 µL Tween 20) pH 6.0). Primary antibodies used to visualize inflammatory processes included cluster of differentiation 68 (CD68, Abcam, cat. no. ab125212), inducible nitric oxide synthase (iNOS, Abcam, cat. no. ab15323), transcription factor interferon regulatory factor 5 (IRF-5, Santa Cruz Biotechnology, cat. no. sc56714), cluster of differentiation 206 (CD206, Abcam, cat. no. ab64693), Arginase-1 (Arg-1, Proteintech Group, cat. no. 16001-1-AP), and the Lymphatic vessel endothelial hyaluronan receptor 1 (LYVE1, Abcam, cat. no. ab33682). As a protein also expressed on endothelial cells of lymphatic vessels, LYVE1 additionally served as a key marker for identifying the newly formed lymphatic network within the regenerated tissue. Vascularization within the regenerated tissue was assessed using cluster of differentiation 31 (CD31, Abcam, cat. no. ab28364), von Willebrand factor (vWF, (ready-to-use, Dako, Agilent, cat. no. IR52761-2), Angiopoeitin1 (Ang1, Abcam, cat. no. ab102015) and alpha smooth muscle actin (α-SMA, Abcam, cat. no. ab7817). The latter also served as a marker for the degree of fibrosis by labelling myofibroblasts within and surrounding the porous scaffold. The ECM was further assessed by staining for Collagen type 1 (Col-1, Abcam, cat. no. ab138492) and Cathepsin K (CathK, Santa Cruz Biotechnology, cat. no. sc-48353). Perilipin1 (Abcam, cat. no. ab3526) was employed as a marker for identifying vital adipocytes within the regenerated tissue. The specificity and validation of IHC stainings were ensured by including positive and negative controls in each experimental staining procedure (Supplementary Figure S6). Negative controls were established using a “no primary antibody” control. Tissue used for positive controls was exclusively derived from porcine sources and included spleen for iNOS, CD206, CD68, and Arg-1; skin for Col-1, Perilipin-1, CD31, Ang-1, and vWF; lymph node for LYVE1 and IRF-5; kidney for CathK; and heart for α-SMA.

## Data Availability

The original contributions presented in the study are included in the article/Supplementary Material, further inquiries can be directed to the corresponding authors.
