# Supplementary material for: Investigating the foreign body response and regenerative mechanisms in medical-grade polycaprolactone scaffold guided breast reconstruction in a porcine model
Source: Front Bioeng Biotechnol. 2026 Jun 17;14:1788533. doi: 10.3389/fbioe.2026.1788533 (PMC13351527; doi:10.3389/fbioe.2026.1788533)
Supplement: Supplementary file 1 [file DataSheet1.pdf]

Supplementary Material

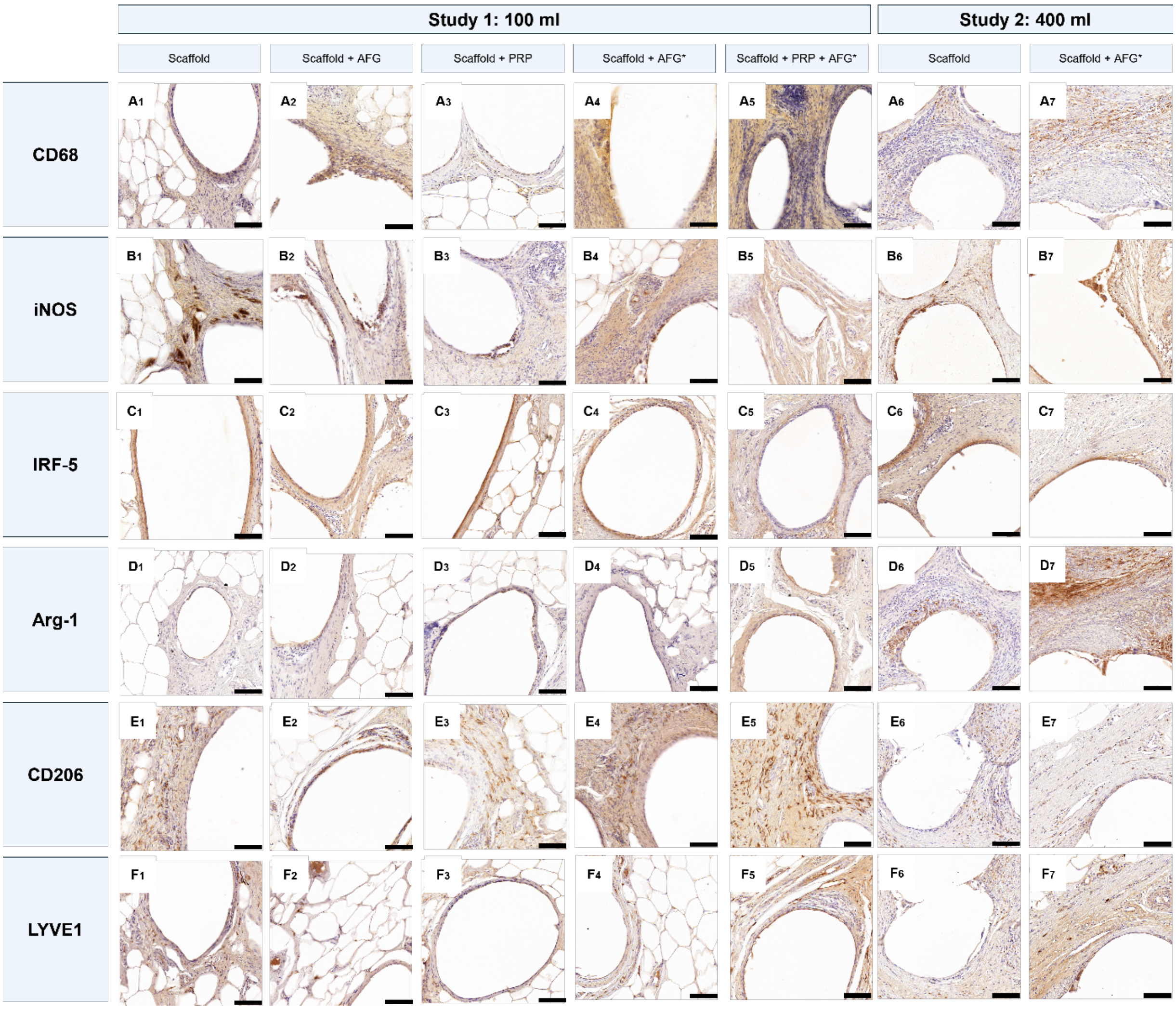

Supplementary Figure S1: Immunohistochemical visualization of the immune response to porous mPCL breast scaffolds 12 months after implantation in a porcine model. Scaffolds of 100 mL (Study 1) and 400 mL (Study 2) volume are shown, stratified by treatment group. (A1–F1) 100 mL scaffolds without adjunctive therapy. (A2–F2) 100 mL scaffolds with immediate fat grafting. (A3–F3) 100 mL scaffolds with immediate platelet-rich plasma (PRP) filling. (A4–F4) 100 mL scaffolds with delayed fat grafting at 4 weeks. (A5–F5) 100 mL scaffolds with immediate PRP filling and delayed fat grafting at 4 weeks. (A6–F6) 400 mL scaffolds without adjunctive therapy. (A7–F7) 400 mL scaffolds with delayed fat grafting at 2 weeks. For each group, the following immunohistochemical stainings were performed: CD68, iNOS, IRF-5, Arg-1, CD206, and LYVE-1. AFG: Autologous fat graft. PRP: Platelet-rich plasma. Scale bar: 100 µm.

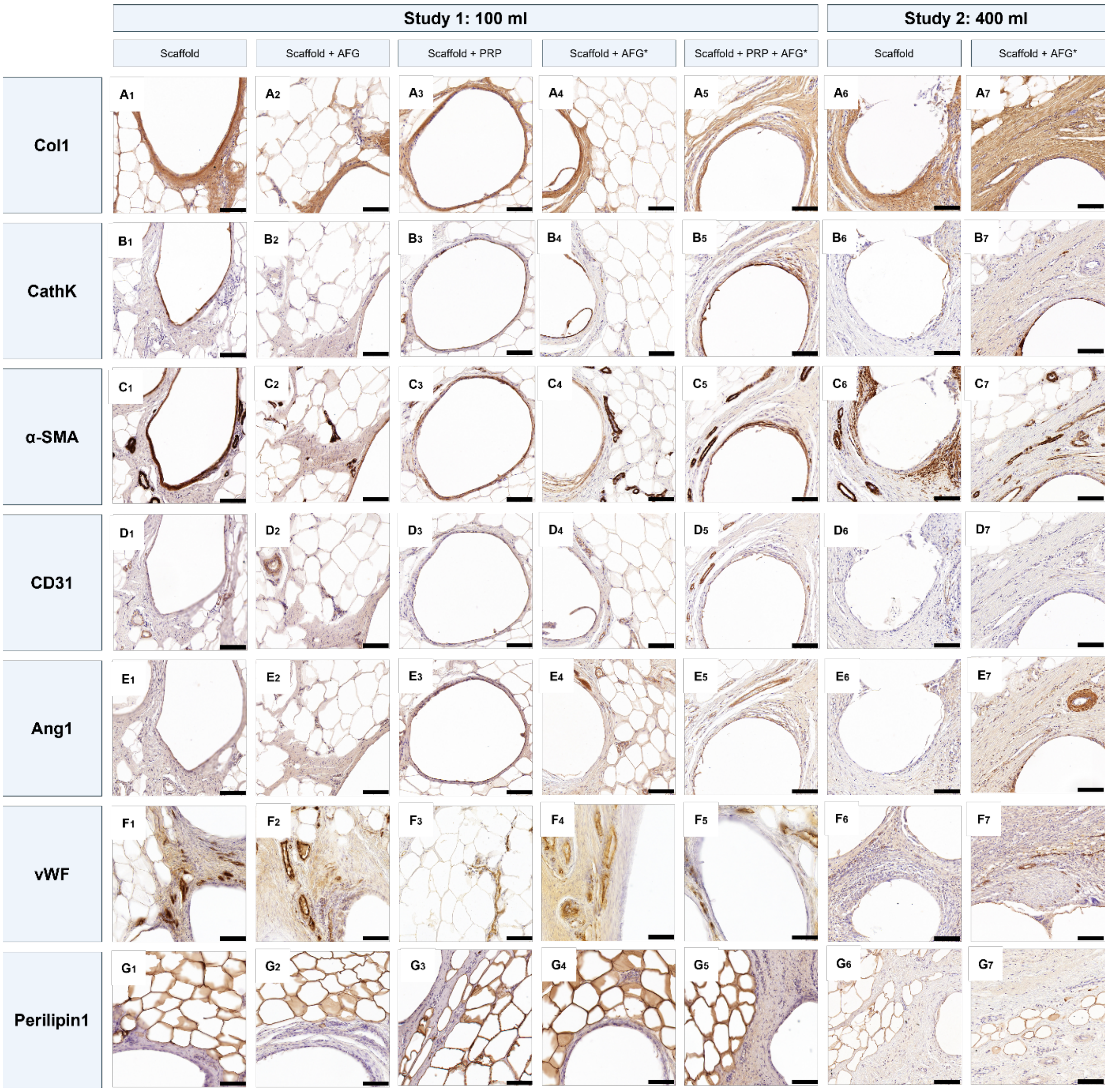

Supplementary Figure S2: **Immunohistochemical visualization of ECM formation, vascularization, and fat regeneration to porous mPCL breast scaffolds 12 months after implantation in a porcine model.** Scaffolds of 100 mL (Study 1) and 400 mL (Study 2) volume are shown, stratified by treatment group. (A1–G1) 100 mL scaffolds without adjunctive therapy. (A2–G2) 100 mL scaffolds with immediate fat grafting. (A3–G3) 100 mL scaffolds with immediate platelet-rich plasma (PRP) filling. (A4–G4) 100 mL scaffolds with delayed fat grafting at 4 weeks. (A5–G5) 100 mL scaffolds with immediate PRP filling and delayed fat grafting at 4 weeks. (A6–G6) 400 mL scaffolds without adjunctive therapy. (A7–G7) 400 mL scaffolds with delayed fat grafting at 2 weeks. For each group, the following immunohistochemical stainings were performed: Col1, CathK, α-SMA, CD31, Ang1, vWF, Perilipin1. AFG: Autologous fat graft. PRP: Platelet-rich plasma. Scale bar: 100 µm.

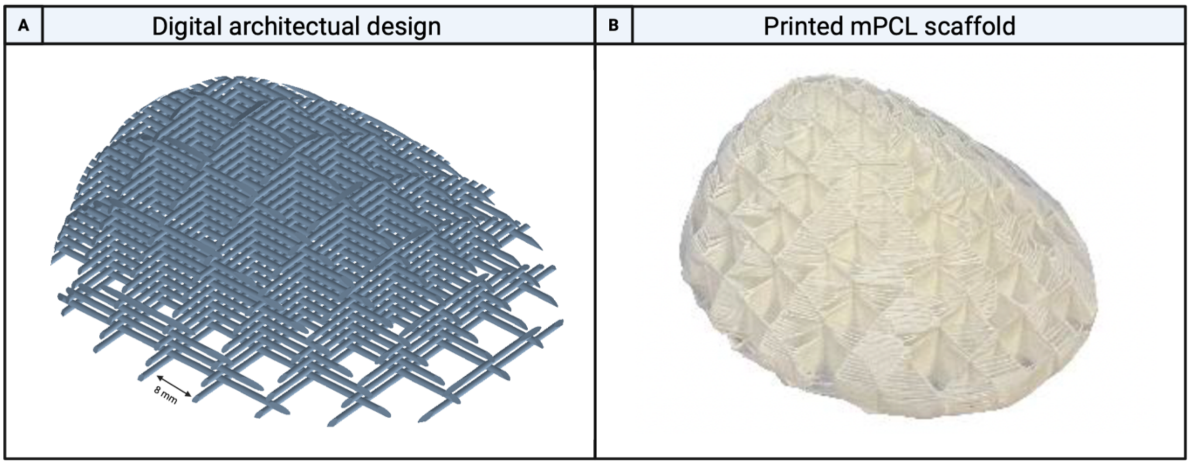

Supplementary Figure S3: Computer-aided design (CAD) of the 100 mL mPCL scaffold used for scaffold-guided breast reconstruction (SGBR) (A) and a photograph (B) of the additively manufactured mPCL scaffold in its breast-shaped configuration. The images highlight the large 8 mm pores and complete (100%) pore interconnectivity. They illustrate the relevance of the selected 0–90° laydown pattern, characterized by open x- and z-directions and a relatively “closed” y-direction, resulting in a channel-like pore architecture designed to guide and support tissue formation.

Supplementary Table S1. Treatment groups used for study 1 and 2.

**Study**		**Group**	**Group description**
**Study 1 (100mL scaffold)**	1	Scaffold	Scaffold only implanted (control)
	2	Scaffold + AFG	Scaffold implanted and filled with 50 mL immediate autologous fat graft (AFG)
	3	Scaffold + PRP	Scaffold implanted and filled with 50 mL immediate platelet-rich plasma (PRP)
	4	Scaffold + AFG*	Scaffold implanted and filled with 50 mL delayed fat graft after four weeks
	5	Scaffold + PRP + AFG*	Scaffold implanted and filled with 50 mL immediate PRP, then 50 mL delayed AFG after four weeks
**Study 2 (400mL scaffolds)**	1	Scaffold	Scaffold only implanted (control)
	2	Scaffold + AFG*	Scaffold implanted and filled with 50 mL delayed fat graft after two weeks

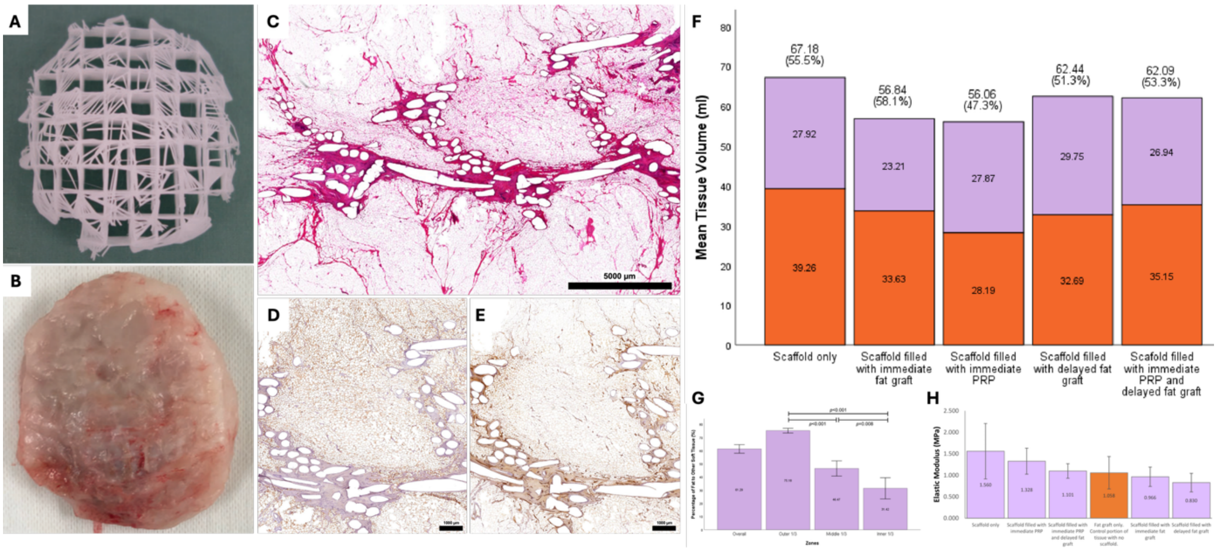

Supplementary Figure S4: Additively manufactured 100 mL breast-shaped porous scaffold composed of medical-grade mPCL. (A) Pre-implantation view of the scaffold before surgical insertion. (B) Explanted construct after 12 months of in vivo implantation in a porcine model. (C) Representative H&E-stained section of regenerated tissue within the scaffold. (D, E) Immunohistochemical staining demonstrating adipose tissue formation (Perilipin-1, D) and vascular structures (von Willebrand factor, E). (F) Quantitative analysis of adipose tissue proportion relative to other tissue components across treatment groups (n = 11 per group), showing the highest fat proportion in the scaffold + immediate fat graft group compared with scaffold + immediate platelet-rich plasma (PRP), without statistically significant intergroup differences (Kruskal–Wallis test). (G) MRI-based regional analysis of adipose tissue distribution within scaffold zones (outer, middle, and inner thirds; n = 43 per zone), demonstrating significant zonal differences (Welch's ANOVA with Games–Howell post hoc test). (H) Mechanical characterization of explanted regenerated tissue constructs after 12 months in vivo. Figures A and B used with permission of Mary Ann Liebert Inc., from A Preclinical Animal Model for the Study of Scaffold-Guided Breast Tissue Engineering, Cheng, Matthew; Janzekovic, Jan; Mohseni, Mina; Medeiros Savi, Flavia; McGovern, Jacqui; Galloway, Graham; Wong, Clement; Saifzadeh, Siamak; Wagels, Michael; Hutmacher, Dietmar W., vol.27, issue 6, 2021; permission conveyed through Copyright © 2021, Mary Ann Liebert, A Division of Sage Publications[1]. Figures C – H modified with permission from Cheng *et. al* [2].

Supplementary information on the methodology of Supplementary Figure S4: Cheng et al.[1,2] evaluated adipose tissue regeneration using 100 mL volume additively manufactured bioresorbable scaffold implanted under the panniculus carnosus muscle along the flanks of twelve pigs over twelve months. Briefly, breast scaffolds were additively manufactured using medical-grade polycaprolactone (mPCL) (8mm pore size measuring 70 x 82 x 44 mm). mPCL scaffolds were plasma treated to improve its hydrophilicity and sterilized by 25 kGy gamma radiation. Under general anaesthetic, pockets were made on each flank of the animal for a total of six treatment sites per animal. These pockets were made in a layer between the panniculus carnosus and deep fascia. Experimental groups: 1) Empty Scaffold, 2) Fat graft only, 3) Scaffold with immediate fat graft, 4) Scaffold with immediate platelet rich plasma (PRP), 5) Scaffold with delayed fat graft, 6) Scaffold with delayed fat graft and PRP.

Autologous fat grafting (AFG): At each designated site, a 1 cm skin incision was made to permit atraumatic cannula insertion. A tumescent solution consisting of 20 ml 0.5% bupivacaine with 1 mg adrenaline diluted in 1 L sterile saline was prepared, and approximately 500 ml was infiltrated evenly across the four abdominal quadrants using a water-jet infiltration cannula in a multilayered, multidirectional fashion to ensure homogeneous tissue distribution, hydrodissection, and hemostasis. Suction-assisted lipoharvesting was then performed with a water-jet-assisted liposuction cannula advanced through the subcutaneous layer using controlled multidirectional back-and-forth movements under continuous manual palpation to avoid skin or abdominal cavity perforation and maintain harvesting within the subdermal adipose compartment until 100 ml of adipose tissue had been collected. Incisions were subsequently closed with absorbable sutures. Collected lipoaspirate was purified by aspirating excess infiltration fluid and blood from the collection chamber via negative pressure after reconnecting the suction system to the irrigation line. The fat graft was then withdrawn with an extraction cannula into a 50 ml syringe and mechanically homogenized by approximately ten repeated transfers between 50 ml and 10 ml syringes connected through a female-to-female Luer-lock adapter, producing controlled emulsification. The processed graft was transferred into capped 10 ml syringes and stored vertically with the outlet downward until implantation. For scaffold filling, syringes containing processed AFG were connected to a needle and inserted through the basal scaffold aspect. Five milliliters were injected during gradual needle withdrawal to create a linear

deposit, followed by four additional fan-shaped radial deposits through the same entry point to achieve homogeneous intrascaffold distribution. This ribbon-like technique was repeated until the predefined total volume had been delivered.

Platelet-Rich Plasma (PRP): Using a multi-lumen central venous catheter, 350 mL of whole blood was aspirated using 20 mL syringes. The collected whole blood was then transferred into the Angel System for PRP preparation, and a processing cycle was performed at a 15% hematocrit setting. Following centrifugation, the PRP fraction was extracted into a 50 mL syringe. If the harvested PRP volume was less than 50 mL, platelet-poor plasma was added until a final total volume of 50 mL was achieved. For activation, a 50 mL syringe containing 10 mL of 10% calcium chloride solution was connected to the PRP-filled syringe. The PRP and calcium chloride solution were then transferred back and forth approximately ten times to ensure homogeneous mixing, after which the activated preparation was evenly distributed into two syringes containing 30 mL each. The addition of calcium chloride induces gelation of PRP. Therefore, to facilitate injection into the allocated scaffold groups, activation was performed immediately prior to injection. Scaffold filling was performed in the same manner as described for the intrascaffold application of AFG.

Mechanical testing: Mechanical testing was conducted to characterize the biomechanical properties of the regenerated soft tissue constructs. Explanted samples were immersed in phosphate-buffered saline (pH 7.0) at 37 °C and subjected to compression using an Instron 5848 MicroTester (Instron, Norwood, MA, USA) at a strain rate of 0.1 mm/s. Samples were compressed to 50% strain, and the resulting stress–strain data were used to estimate the elastic modulus of the constructs.
Imaging: In vivo imaging was performed using computed tomography (CT) to assess the volume and spatial distribution of regenerated tissue over time. Animals were anesthetized or sedated to minimize motion artifacts and ensure high-quality image acquisition. Each animal was positioned in dorsal recumbency on the CT table, with precise alignment achieved via the scanner's laser positioning system. Animals were stabilized with towels and straps, and table height was adjusted to ensure central positioning. Imaging was conducted by certified personnel following standard radiation safety protocols, including the use of lead protective equipment and controlled access to the imaging suite. Immediately post-euthanasia, CT scans (Toshiba Aquilion

Lightning, Tokyo, Japan) were acquired using standard helical acquisition prior to scaffold explantation. Explanted scaffolds were rescanned by CT at 0.5 mm slice thickness, followed by MRI on a 3T system (Siemens MAGNETOM Prisma, Munich, Germany) employing T1- and T2-weighted fat-suppressed sequences and water/fat spectral excitation for tissue differentiation. Volumetric analysis of CT data was performed using the open-source software Horos 4.0.

Histological analysis included H&E, Masson's Trichrome staining, and immunohistochemical saining for Perilipin (PLN1) and von Willebrand Factor (vWF) antibodies.

Histomorphometry: Distribution analysis was performed using ImageJ 1.54i. Fat saturated sequences were used, and a middle slice through the scaffold construct was imported. This slice was divided into three zones and converted to a binary scale. The proportion of high signal, corresponding to fat, and low signal, corresponding toother tissue types, was calculated in each zone.

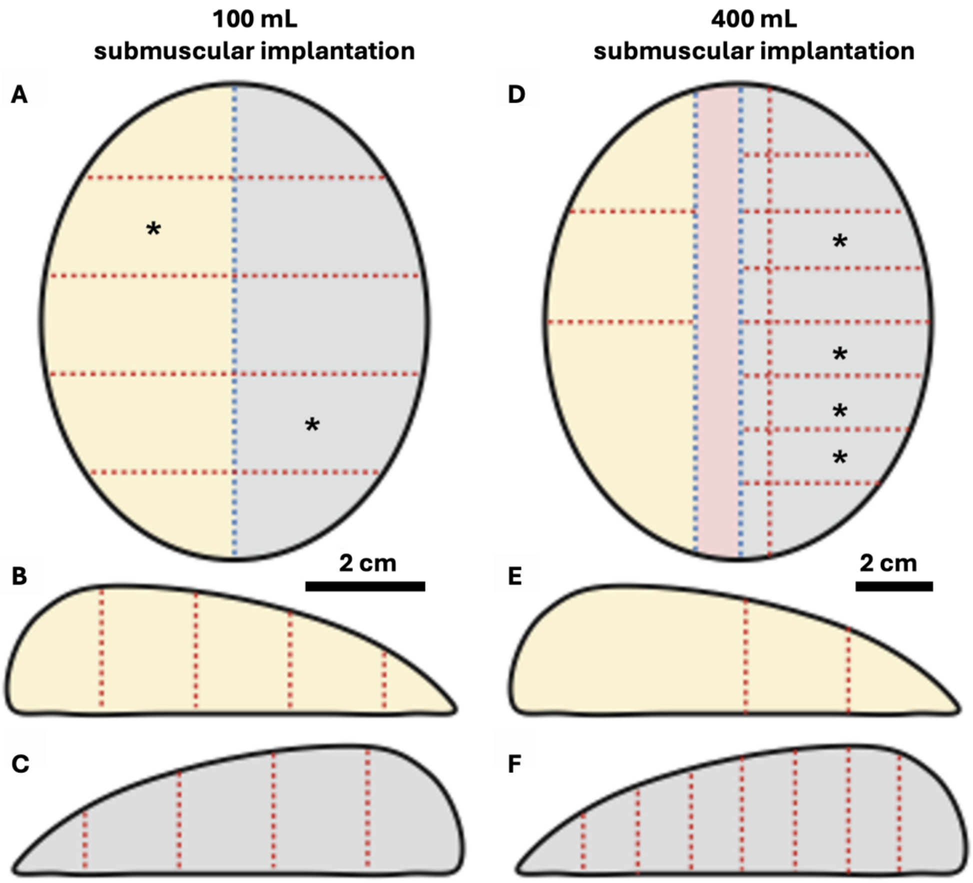

Supplementary Figure S5: Schematic illustration of the subsectioning pattern applied to the explanted scaffolds after 12 months in vivo. The scaffolds were sectioned into multiple 1–2 cm tissue blocks, as shown in the top view for the 100 mL scaffold (A) and the 400 mL scaffold (D). The corresponding lateral views are presented for the 100 mL scaffold (B, C) and the 400 mL scaffold (E, F) respectively. Sections used for histological analysis are indicated with an (*).

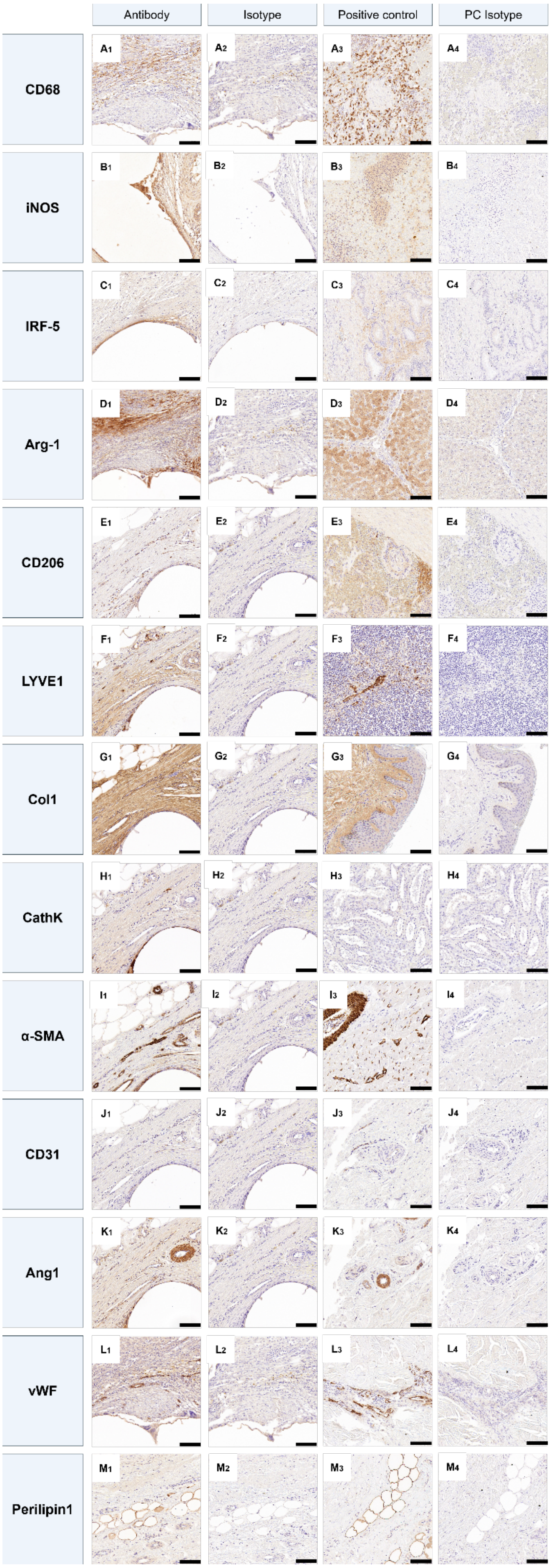

Antibody
Isotype
Positive control
PC Isotype
CD68
iNOS
IRF-5
Arg-1
CD206
LYVE1
Col1
CathK
α-SMA
CD31
Ang1
vWF
Perilipin1
A1 A2 A3 A4
B1 B2 B3 B4
C1 C2 C3 C4
D1 D2 D3 D4
E1 E2 E3 E4
F1 F2 F3 F4
G1 G2 G3 G4
H1 H2 H3 H4
I1 I2 I3 I4
J1 J2 J3 J4
K1 K2 K3 K4
L1 L2 L3 L4
M1 M2 M3 M4

Supplementary Figure S6: Immunohistochemical validation of primary antibodies. Representative stainings for each antibody are shown (A1–M1), alongside corresponding no-primary-antibody negative controls (A2–M2). Positive control tissues are displayed for each marker (A3–M3), with matched no-primary-antibody negative controls for the positive controls (A4–M4). All positive control tissues were derived exclusively from porcine specimens. The following tissues were used for validation: CD68 (spleen), iNOS (spleen), IRF-5 (liver), Arg-1 (liver), CD206 (spleen), LYVE-1 (lymph node), collagen I (skin), cathepsin K (kidney), α-SMA (myocardium), CD31 (skin), angiopoietin (skin), von Willebrand factor (skin), and perilipin-1 (skin). Scale bar: 100 µm.

References

(1) Cheng, M.; Janzekovic, J.; Mohseni, M.; Medeiros Savi, F.; McGovern, J.; Galloway, G.; Wong, C.; Saifzadeh, S.; Wagels, M.; Hutmacher, D. W. A Preclinical Animal Model for the Study of Scaffold-Guided Breast Tissue Engineering. *Tissue Eng. Part C Methods* **2021**, *27* (6), 366–377. https://doi.org/10.1089/ten.TEC.2020.0387.

(2) Cheng, M.; Janzekovic, J.; Finze, R.; Mohseni, M.; Saifzadeh, S.; Savi, F. M.; Ung, O.; Wagels, M.; Hutmacher, D. W. Conceptualizing Scaffold Guided Breast Tissue Regeneration in a Preclinical Large Animal Model. *Bioengineering* **2024**, *11* (6), 593. https://doi.org/10.3390/bioengineering11060593.
